# Non-tumoral uptake of ^68^Ga-FAPI-04 PET: A retrospective study

**DOI:** 10.3389/fonc.2022.989595

**Published:** 2022-12-01

**Authors:** Na Qi, Hao Wang, Haiyan Wang, Shuhua Ren, Zhiwen You, Xing Chen, Yihui Guan, Fang Xie, Fengchun Hua, Jun Zhao

**Affiliations:** ^1^ Department of Nuclear Medicine, Shanghai East Hospital, Tongji University School of Medicine, Shanghai, China; ^2^ Department of General Surgery, Huashan Hospital, Fudan University, Shanghai, China; ^3^ PET Center, Huashan Hospital, Fudan University, Shanghai, China; ^4^ Department of Nuclear Medicine, Longhua Hospital, Shanghai University of Traditional Chinese Medicine, Shanghai, China

**Keywords:** 68 Ga-FAPI-04, SUV, physiological uptake, benign uptake, multicenter retrospective study

## Abstract

**Objective:**

Fibroblast activation protein (FAP)-targeting radiopharmaceutical based on the FAP-specific inhibitor (FAPI) is considered as a potential alternative agent to FDG for tumor-specific imaging. However, FAP is also expressed in normal adult tissues. The aim of this study was to explore the image features of non-tumoral regions with high uptake of ^68^Ga-FAPI-04 in positron emission tomography (PET) imaging and to reveal the physiological mechanisms of these regions.

**Material:**

A total of 137 patients who underwent whole-body ^68^Ga-FAPI-04 PET/MR (n=46) or PET/CT (n=91) were included in this retrospective study. Three experienced nuclear medicine physicians determined the non-tumoral regions according to other imaging modalities (CT, MRI, ^18^F-FDG PET, or ultrasound), clinical information, or pathological results. The regions of interest (ROIs) were drawn manually, and the maximum standardized uptake value (SUV_max_) was measured.

**Results:**

A total of 392 non-tumoral uptake regions were included in this study. The included physiological regions were uterus (n=38), submandibular gland (n=118), nipple (n=37), gingiva (n=65), and esophagus (n=31). The incidence of ^68^Ga-FAPI-04 uptake in physiological regions was independent of age, the tracer uptakes in the gingiva and esophagus were more common in male patients (*p*=0.006, 0.009), while that in the nipple was more common in female patients (*p* < 0.001). The included benign regions were inflammatory lymph node (n =10), pneumonia (n=13), atherosclerosis (n=10), pancreatitis (n=18), osteosclerosis (n=45), and surgical scar (n=7). No significant difference was observed in SUV_max_ between physiological and benign regions.

**Conclusions:**

A number of organs exhibit physiological uptakes of ^68^Ga-FAPI-04. Our study showed that regions with high ^68^Ga-FAPI-04 uptake did not necessarily represent malignancy. Being familiar with physiological and typical benign ^68^Ga-FAPI-04 uptake regions can be helpful for physicians to interpret images and to make an accurate diagnosis.

## Introduction

Cancer-associated fibroblasts (CAFs) and extracellular fibrosis can account for 90% of the total tumor mass (1). Fibroblast activation protein (FAP), a type II membrane-bound glycoprotein of the dipeptidyl peptidase 4 family, is over-expressed in CAFs of many epithelial carcinomas and is involved in a variety of tumor-promoting activities, such as stromal remodeling, angiogenesis, chemotherapy resistance, and immunosuppression (1, 2). Since FAP is expressed at low levels in most normal organs, it is a promising target for imaging and radiation therapy (3). Radiopharmaceuticals targeting FAP have recently been developed based on FAP-specific inhibitors (FAPIs) (4). Among several recently developed tracers targeting FAP, ^68^Ga-FAPI-04 is regarded as a promising one for having high affinity towards FAP and suitable kinetics (5–7). Without the necessity of fasting in preparation before the scan and an equal or better tumor-to-background ratio compared with ^18^F-FDG PET scans, ^68^Ga-FAPI-04 is considered as a potential alternative agent to FDG for tumor-specific imaging (8).

Currently, most FAPI studies are focused on tumor imaging. Besides its high expression in epithelial carcinoma (8), FAP also plays a key role in normal development during embryo-genesis and tissue modeling (9). FAP can also be expressed in normal adult tissues such as active tissue damage, remodeling, inflammation, arthritis, atherosclerotic plaques, and fibrosis (3, 9, 10). Several non-oncology studies on FAPI revealed its unique values in IgG4-related diseases (11). Luo et al. (12) found that compared with ^18^F-FDG, ^68^Ga-FAPI-04 was more effective in detecting organs affected by IgG4-related disease. An animal study showed that joint FAPI concentration was correlated with arthritis scores in rats (13). A recent work reported that ^68^Ga-FAPI-04 focal non-tumoral uptake can occur in fibrous lesions, fibrous hyperplasia, and fibrous activity (14). ^68^Ga-FAPI-04 could also accumulate in some benign diseases of the bones and joints (15). Recent studies characterized the benign lesions with increased ^68^Ga-FAPI-04 uptake in PET/CT (16, 17). However, to the best of our knowledge, there are no systematic studies to reveal the pathophysiological mechanisms of non-tumoral ^68^Ga-FAPI-04 uptake regions. This study aimed to investigate the uptake characteristics in non-tumoral regions using ^68^Ga-FAPI-04 PET/CT or PET/MR with a relatively large sample size and provide a reference for imaging diagnosis.

## Materials and methods

### Patients

This retrospective analysis was performed on patients who underwent ^68^Ga-FAPI-04 PET/CT (Biograph mCT, Siemens Healthineers, Germany; Ingenuity TF, Philips Healthcare, USA; uMI510, United Imaging, China) or ^68^Ga-FAPI-04 PET/MR (uPMR790 TOF, United Imaging, China) from April 2020 to August 2021. The inclusion criteria were as follows: (i) patients who were able to sign informed consents for examination according to the guidelines of the Clinical Research Ethics Committee; (ii) patients with a predicted survival of more than 6 months. Exclusion criteria were (i) pregnancy, (ii) postmenopausal women with taking hormone replacement or related drugs, and (iii) patients with a predicted survival of <6 months.

### Radiopharmaceutical and imaging protocols

Good-manufacturing-practice (GMP)-grade precursors ^68^Ga-FAPI-04 was synthesized in the Radiochemistry Facility of the PET Center, Huashan Hospital, Fudan University, according to the protocol described previously (18). The radiochemical purity of ^68^Ga-FAPI-04 was over 95%, and the final product was sterile and pyrogen-free.

Whole-body PET/CT or PET/MR scans were performed 60 min after the injection of ^68^Ga-FAPI-04 with a dose of 150 ± 35 MBq (4.05 ± 0.95 mCi) from the vertex to the mid-thigh. For PET/CT, a PET scan was acquired after a low-dose CT scan, which was performed at 120 kV and 100–120 valid mAs. Brain PET scanning was performed 5 min/bed, and body PET scanning was performed 3 min/bed. PET/MR was performed with default clinical MRI sequences including T1w and T2w (TE = 2.24 ms, TR = 4.91 ms, flip angle = 10, echo train length = 30, FOV = 549 × 384, matrix = 256 × 329, slice thickness = 2 mm, slice spacing = 2 mm, transverse plane) (18). PET images were reconstructed by ordered subset expectation maximization 3D (OSEM 3D) method with 2 iterations and 20 subsets.

Since different scanners were used in this study, SUV measurements were normalized after data collection. A NEMA IEC body phantom (Data Spectrum Corporation, Durham, NC, USA) with six simulated lesion spheres (diameters: 10, 13, 17, 22, 28, and 37 mm) was applied for SUV normalization with 2, 4, 8, and 16 times the background activity (background activity concentration =2 kBq/ml). A CT scan of the NEMA IEC body phantom was prepared for the attenuation correction of PET/MR. Correlation coefficients were obtained through this phantom study and used to standardize the SUV measurements as previously reported (18, 19).

### PET/CT and PET/MR imaging review

Three nuclear medicine physicians with 15, 10, and 8 years of experience in interpreting PET/CT and MR imaging determined the physiological and benign tracer uptake regions based on the patients’ clinical data, imaging data (CT, MRI, ^18^F-FDG PET, or ultrasound), histopathology, and their own experiences in image interpretation. Physiological uptake refers to the slightly elevated uptake of ^68^Ga-FAPI-04 in generally normal tissues, which usually show no abnormal changes on other imaging modalities (20, 21). Benign uptake refers to inflammation, fibrosis, benign tumors, and other non-malignant tumor regions that may be abnormal on other imaging modalities (16, 22). For any differences in opinion, a consensus was reached by discussion together. The ROI was drawn manually for SUV_max_ measurement.

### Statistical analyses

Shapiro–Wilk normality test was used to analyze the data distribution. Data were expressed as mean ± standard deviation (SD). Data of physiological and benign uptake regions were tested by independent sample T-test. Chi-square test and logistic regression analysis were used to investigate the influence of age and sex on the incidence of physiological uptake regions. Pearson correlation analysis was performed for SUV_max_ of physiological regions and age. Statistical analysis was performed using SPSS 23.0 statistical software. Two-tailed *p* < 0.05 was considered statistically significant.

## Results

### Patient characteristics

Patient characteristics are summarized in **Table 1**. Briefly, 137 patients (84 male and 53 female; age, 58 ± 14 years; range from 18 to 86 years, mostly diagnosed with cancer) were included in this study. Non-tumoral regions were observed in the majority of patients (86.86%). A total of 392 non-tumoral regions were classified as physiological regions (n = 289, SUV_max_ = 3.62 ± 2.86) or benign regions (n = 103, SUV_max_ = 3.50 ± 2.25) according to other imaging features, clinical representations, or pathological results. T-test indicated no statistically significant difference between the physiological and benign groups (*p* = 0.40).

**Table 1:** Patient characteristics.

N = 137	Overall
Age	
Mean (SD)	58 (14)
Median [min, max]	60 [18,86]
Sex	
Male	84
Female	53
Diagnosis	
Liver cancer	38
Gastric cancer	36
Gynecological cancer	11
Lung cancer	7
Colorectal cancer	6
Pancreatic cancer	5
Neuroendocrine tumor	2
Duodenal tumor	2
Renal cancer	2
Breast cancer	1
Osteosarcoma	1
Esophagus cancer	1
Lymphoma	1
Prostate cancer	1
Other	23

### Physiological uptake regions

**Figure 1:**
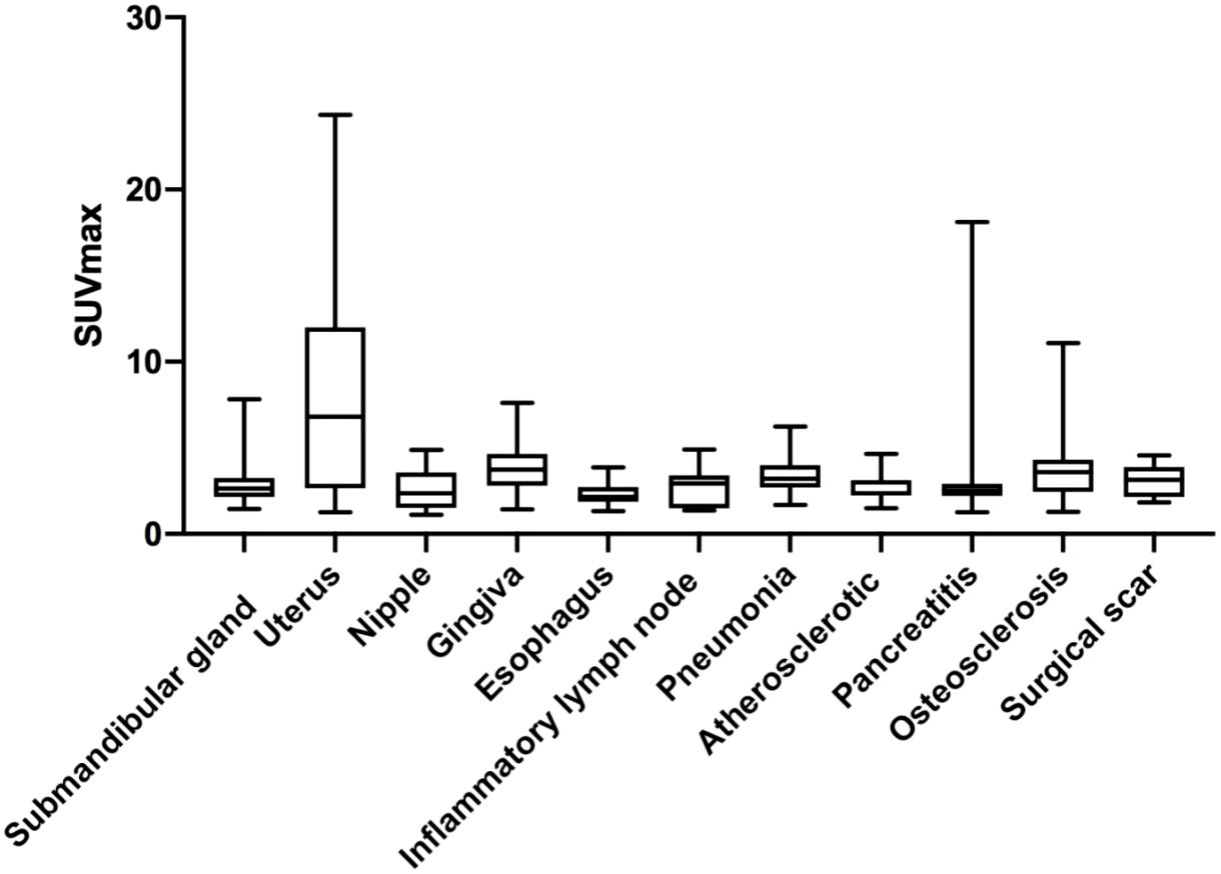
SUV_max_ of non-tumoral tracer uptake regions.

The physiological uptakes are summarized in **Table 2** and **Figure 1**. Elevated ^68^Ga-FAPI-04 uptakes in the head and neck were primarily observed in the submandibular gland (n = 118, SUV_max_ range from 1.46 to 7.83) and gingiva (n = 65, SUV_max_ range from 1.43 to 7.61), while in the chest, elevated uptake was mainly located in the nipple (n = 37, SUV_max_ range from 1.12 to 4.88) and esophagus (n = 31, SUV_max_ range from 1.33 to 3.87). Although the incidence of ^68^Ga-FAPI-04 uptake in the submandibular gland, gingiva, nipple, and esophagus was independent of age (*p*>0.05), the tracer uptakes in the gingiva and esophagus were more common in male patients (*p* = 0.006, 0.009), while uptakes in the nipple were more common in female patients (*p*<0.001) (**Table 3**). The uptake values of ^68^Ga-FAPI-04 (SUV_max_) in the submandibular gland were positively correlated with age (*p* = 0.01) and higher in male patients (*p* = 0.001), while those in other physiological regions were independent of age and sex (all *p* ≥ 0.05) (**Table 3**).

**Table 2:** Physiological uptake of ^68^Ga-FAPI-04.

	Incidence (%)			SUVmax								
	All	Male	Female	All			Male			Female		
Submandibular gland	45.26	51.19	35.85	2.81 ± 0.97			3.01 ± 1.05			2.33 ± 0.52		
Gingiva	47.45	57.14	32.08	3.82 ± 1.33			3.94 ± 1.31			3.47 ± 1.36		
Nipple	14.6	3.57	32.08	2.6 ± 1.17			1.94 ± 0.53			2.73 ± 1.22		
Esophagus	22.63	30.95	9.43	2.31 ± 0.64			2.34 ± 0.59			2.13 ± 0.9		
Uterus	NA	NA	71.7	NA			NA			7.82 ± 5.78		

NA, Not Applicable.

**Table 3:** Main effects of age and sex on the ^68^Ga-FAPI-04 uptake.

	Age			Sex		
	Coef	[95% CI]	*p*	Coef	[95% CI]	*p*
Incidence						
Submandibular gland	0.01	[−0.01, 0.04]	0.31	−0.53	[−1.26, −0.20]	0.16
Gingiva	−0.003	[−0.03, 0.02]	0.8	−1.07	[−1.82, −0.31]	0.006
Nipple	−0.01	[−0.05, 0.03]	0.6	2.47	[1.16, 3.79]	<0.001
Esophagus	0.006	[−0.03, 0.04]	0.75	−1.42	[−2.48, −0.36]	0.009
SUVmax						
Submandibular gland	0.006	[0.001, 0.01]	0.01	−0.21	[−0.32, −0.09]	0.001
Gingiva	−0.02	[−0.05, −0.005]	0.11	−0.64	[−1.40, 0.13]	0.1
Nipple	0.006	[−0.02, 0.03]	0.68	0.84	[−0.23, 1.92]	0.12
Esophagus	0.00	[−0.01, 0.01]	0.94	−0.13	[−0.40, 0.14]	0.34

High uptake of ^68^Ga-FAPI-04 in the uterus was also very common (n=38, mean SUV_max_ = 7.82 ± 5.78, SUV_max_ range from 1.26 to 24.33). Increased ^68^Ga-FAPI-04 uptake in the uterus was observed in 71.70% of female patients and occurred preferentially in premenopausal women (82.14%, *p* = 0.07). The SUV_max_ in the uterus did not correlate with the patients’ age (r = −0.11, *p* = 0.50). When comparing SUV_max_ in the uterus between premenopausal and postmenopausal groups, no statistically significant difference was observed (SUV_max_ = 8.40 ± 5.64 vs. 6.93 ± 6.06, *p* = 0.50).

### Benign uptake regions

The benign regions included inflammatory lymph node (n = 10, mean SUV_max_ = 2.75 ± 1.13, SUV_max_ range from 1.37 to 4.91), pneumonia (n = 13, mean SUV_max_ = 3.37 ± 1.22, SUV_max_ range from 1.68 to 6.24), atherosclerosis (n = 10, mean SUV_max_ = 2.85 ± 0.84, SUV_max_ range from 1.49 to 4.65), pancreatitis (n = 18, mean SUV_max_ = 3.41 ± 3.74, SUV_max_ range from 1.27 to 18.11), osteosclerosis (n = 45, mean SUV_max_ = 3.93± 2.22, SUV_max_ range from 1.28 to 11.09), and surgical scar (n = 7, mean SUV_max_ = 3.14 ± 0.98, SUV_max_ range from 1.83 to 4.56). There was no significant difference in SUV_max_ between these regions (*p*>0.05).

**Figure 2:**
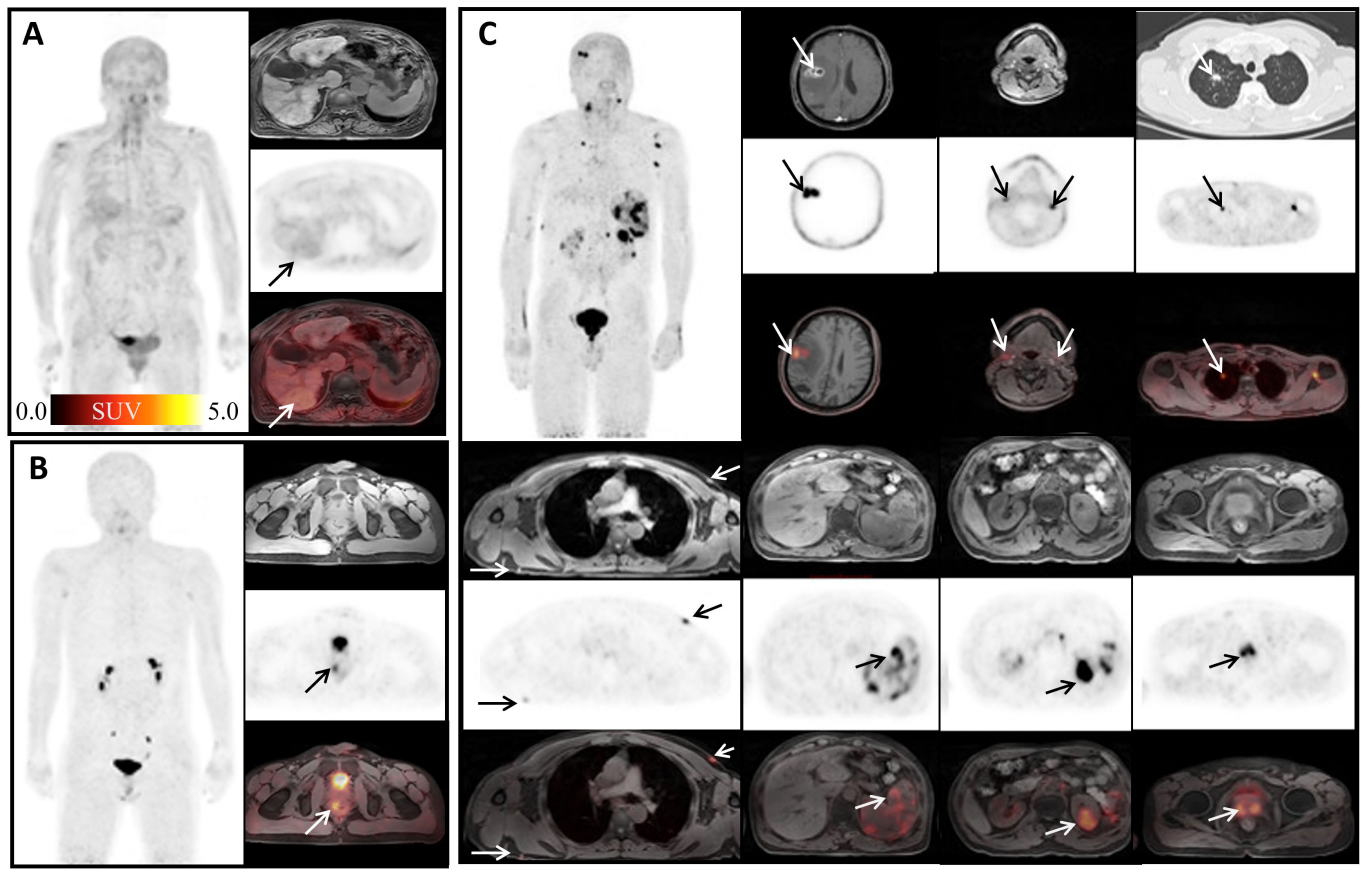
Interesting cases ^68^Ga-FAPI-04 imaging. **(A)** A 65-year-old woman with a history of hepatitis B over 30 years, arrows, cirrhosis of the liver, SUV_max_ 3.24; **(B)** a 19-year-old man with a 2-year history of rectal Crohn’s disease, arrows, rectal Crohn’s disease, SUV_max_ 5.22; **(C)** a 56-year-old man diagnosed with disease of disseminated non-tuberculous mycobacteriosis. The maximum intensity projection (MIP) image shows various FAPI-avid nodules: brain (SUV_max_ =2.61), cervical lymph nodes (SUV_max_ =1.81), upper lobe of right lung (SUV_max_ = 1.74), subcutaneous nodule on the left chest (SUV_max_ = 3.52), spleen (SUV_max_= 2.03), left kidney (SUV_max_ = 2.76), and prostate (SUV_max_ = 4.02).

We found some interesting cases with high ^68^Ga-FAPI-04 uptakes. A patient with a 30-year history of hepatitis B showed high ^68^Ga-FAPI-04 uptake in the liver (**Figure 2A**). High ^68^Ga-FAPI-04 uptake has also been found in the rectum of a patient with Crohn’s disease (**Figure 2B**). A man diagnosed with disseminated non-tuberculous mycobacteriosis (tuber colectomy of the left chest wall and CT-guided percutaneous lung puncture biopsy found inflammatory granulomatous lesions; prostate puncture pathology revealed non-specific granulomatous prostatitis; second-generation DNA sequencing results suggested occasional mycobacterium infection) showed lesions throughout the body with high or mild uptake of ^68^Ga-FAPI-04 (**Figure 2C**). After anti-infective therapy, the intracranial lesions became smaller.

## Discussion

Due to the specific expression of FAP in tumor stromal fibrous tissues, FAP has received increasing attention as a specific marker of CAFs. Meanwhile, activated fibroblasts that undergo extracellular matrix (ECM) remodeling in the tissue due to chronic inflammation, fibrosis, and wound healing can also be observed by FAPI imaging (23–25). In this study, we described the SUV_max_ of 392 non-tumoral uptake regions in 137 patients who underwent ^68^Ga-FAPI-04 PET/CT or PET/MR.

**Figure 3:**
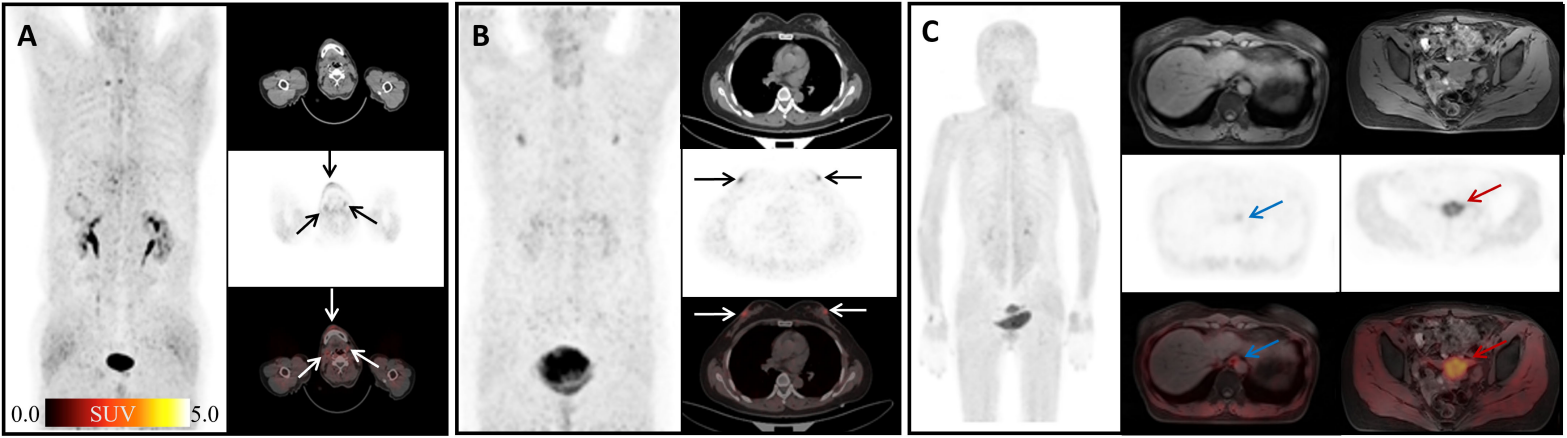
Physiological uptake of ^68^Ga-FAPI-04. **(A)** A 60-year-old man with hepatic hilar malignancy after the treatment of transcatheter arterial chemoembolization (TACE); the arrows indicate ^68^Ga-FAPI-04 uptake in the gingiva and submandibular gland (SUV_max_ 4.45, 3.79, and 3.64). **(B)** A 65-year-old woman with microinvasive lung adenocarcinoma 6 months after surgery; arrows show physiological uptake in the nipples with SUV_max_ 4.88. **(C)** A 55-year-old woman with signet ring cell carcinoma of stomach 2 months after endoscopic submucosal dissection (ESD); blue arrows show physiological uptake of the esophagus (SUV_max_ =3.67), and red arrows show uterus (SUV_max_=13.94).

Consistent with previous studies (7, 26), physiological uptakes of ^68^Ga-FAPI-04 were observed in the submandibular gland, nipple, gingiva, and esophagus (**Figures 3A–C**). In our study, the incidence of ^68^Ga-FAPI-04 uptakes in the submandibular gland, gingiva, nipple, and esophagus were independent of age. Tracer uptakes in the gingiva and esophagus were more common in male patients, whereas uptake in the nipple was more common in female patients. It indicates that sex may have a more significant effect on physiological expression of FAPI than age. The uptake values of ^68^Ga-FAPI-04 (SUV_max_) in the submandibular gland were positively correlated with age, suggesting that FAP activity in the submandibular gland may be affected by age.

The uptake of ^68^Ga-FAPI-04 in the uterus was significantly higher compared to other non-tumoral regions in our study (**Figure 3C**, red arrow). The high uptake in the uterus is considered to stem from the endometrial glandular cells, and its level is significantly lower than that of the malignant component in the uterus (27). Although a recent work suggested that tracer uptake decreases with age (28), in this study, we did not find a significant correlation between the SUV_max_ of uterus and patient age, in line with a previous study (17) reporting that intense ^68^Ga-FAPI-04 uptake in the uterus was independent from menopause. High uterine FAP activity might be caused by tissue remodeling and angiogenesis during hormonal periodic changes in regeneration (29).

**Figure 4:**
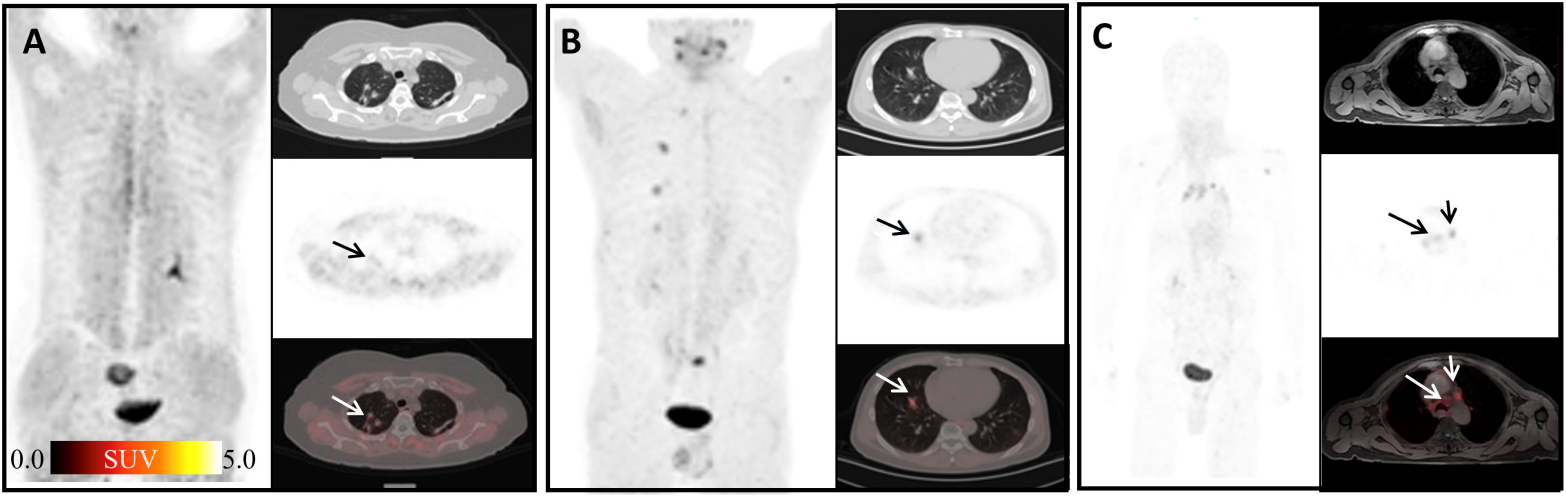
Physiological uptake of ^68^Ga-FAPI-04. **(A)** A 64-year-old female patient with gastric cancer 1 year after surgery; the arrows show organized pneumonia in the upper lobes of right lung with SUV_max_ of 1.76. **(B)** A 60-year-old male patient after liver cancer surgery; the arrows indicate the inflammatory lesion in the middle lobe of the right lung with SUV_max_ 3.24. **(C)** A 50-year-old male patient with weight loss of 10 kg in recent 6 months; arrows show mediastinal inflammatory lymph nodes with SUV_max_ of 4.91.

FAP can be induced by fibrosis foci during pulmonary fibrosis in ongoing tissue remodeling (30). In this study, elevated uptake of ^68^Ga-FAPI-04 was found in 13 pneumonia lesions (mean SUV_max_ = 3.37) (**Figures 4A, B**), yet still lower than that in lung cancer lesions (SUV_max_>12) according to the literature (31). Although ^68^Ga-FAPI-04 PET/CT is inferior to ^18^F-FDG PET/CT in detecting lymph nodes involved in IgG4-related diseases (12), Schmidkonz et al. reported high uptake of FAPI in lymph nodes infiltrated by a fibrotic process and decreased FAPI uptake in those after anti-fibrosis therapy (11). Inflammatory lymph nodes in our study also showed high uptake of ^68^Ga-FAPI-04 (**Figure 4C**), and SUV_max_ was lower than that of fibrotic lymph nodes reported before (11). Mixed type of proliferative and fibrotic lymph nodes in our study may have led to such results.

**Figure 5:**
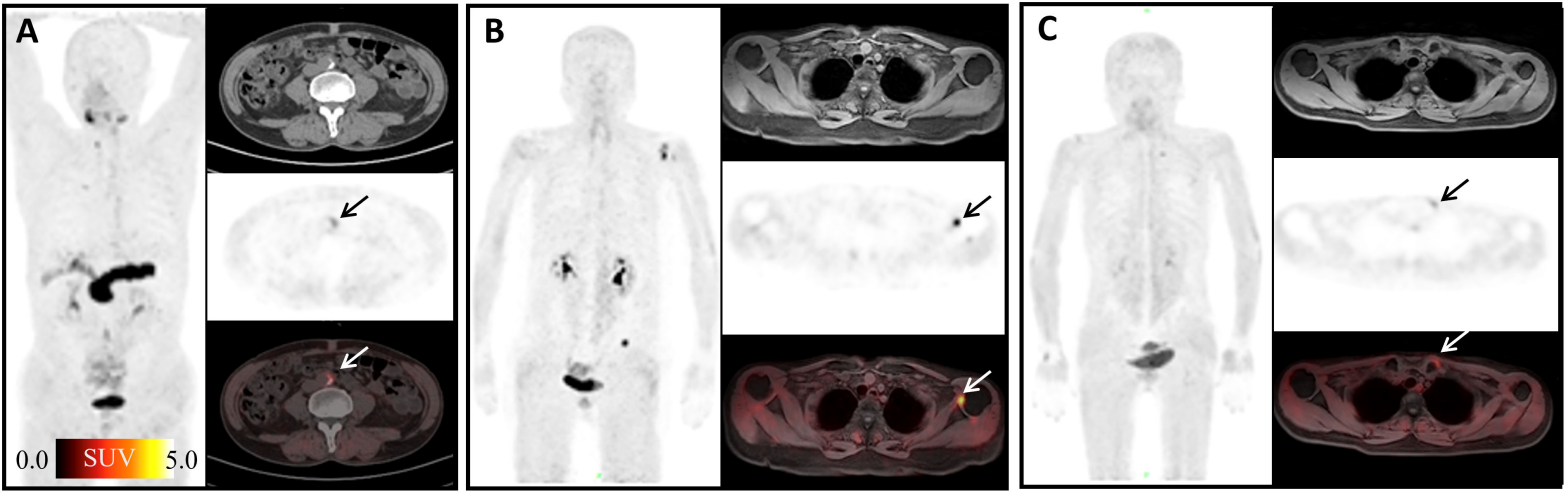
Physiological uptake of ^68^Ga-FAPI-04. **(A)** A 72-year-old man with duodenal papillary tumor; arrows show atherosclerosis of the abdominal aorta with SUV_max_ =4.65. **(B)** A 70-year-old woman presented with adenocarcinoma at the descending colon–sigmoid junction; arrows show left shoulder arthritis with SUV_max_ =11.09. **(C)** A 55-year-old woman with signet ring cell carcinoma of stomach 2 months after ESD; arrows show left sternoclavicular arthritis with SUV_max_ = 8.21.

FAP has recently been proposed as an inflammation-induced protease involved in the formation of vulnerable plaques (32). It has been reported that FAP expression was enhanced in the human atherosclerotic vessel and increased upon plaque progression (33). In our study, atherosclerotic plaques showed slightly high uptake of ^68^Ga-FAPI-04 with mean SUV_max_ = 2.85 (**Figure 5A**). Forty-five joints in our study showed high ^68^Ga-FAPI-04 uptake (**Figures 5B, C**). In a study of the biological distribution of FAPI in cancer patients, mild low-grade uptake in the knee and shoulder was observed in three patients with no clinical symptoms of arthritis (34). FAP expression has been observed in synovial tissue samples of rheumatoid arthritis (35). In osteoarthritis, higher levels of FAP expression on the surface of the cartilage and on chondrocyte membranes were detected by Milner et al. (36). Terry Sy and his colleagues found that In-28H1 (anti-FAP antibody) radionuclide imaging could be used to evaluate the treatment response to etanercept in arthritic mice (13). Therefore, ^68^Ga-FAPI-04 might present a potential therapeutic target of arthritis, and ^68^Ga-FAPI-04 imaging has potential value in diagnosis and therapeutic efficacy evaluation in the future.

**Figure 6:**
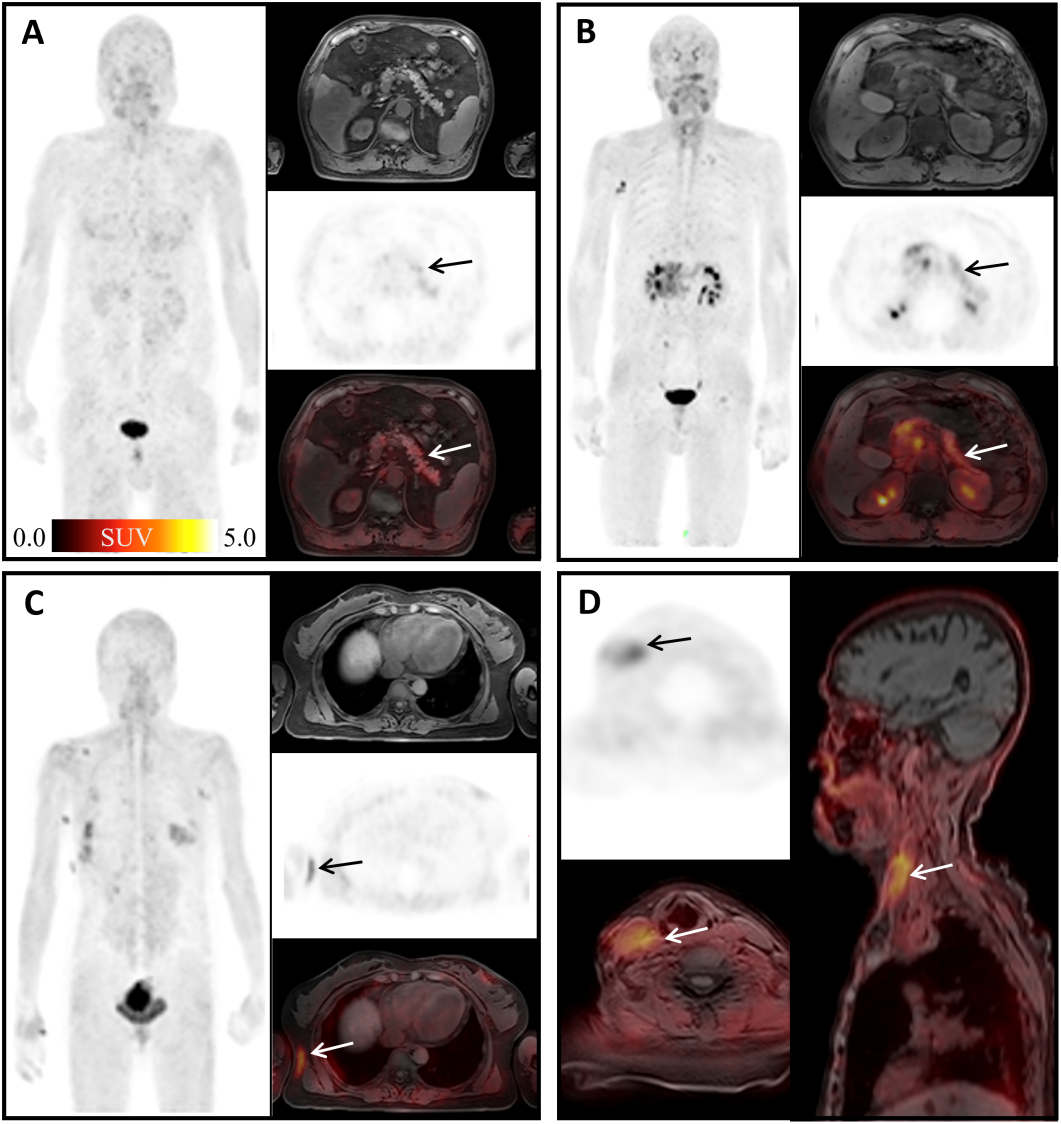
Physiological uptake of ^68^Ga-FAPI-04. **(A)** A 67-year-old man with mucinous adenocarcinoma of the right lung 1 year after surgery; arrows show pancreatic diffuse inflammatory uptake, SUV_max_ =4.66. **(B)** A 63-year-old man diagnosed with duodenal malignancy; arrows show obstructive pancreatitis, SUV_max_ = 18.11. **(C)** A 49-year-old woman, 3 months after surgery for early microinfiltrating adenocarcinoma of the right middle lobe and 2 months after surgery for left breast fibroma; arrows show surgical scar on the right chest with SUV_max_ =3.89. **(D)** An 83-year-old woman diagnosed with gastric cancer 6 months ago; arrows show deep vein catheterization area of the right neck with SUV_max_ =4.56.

It has been reported that ^68^Ga-FAPI-04 could show focal high uptake in pancreatic fibrous lesions, fibroplasia, or fibrotic activity (14). Our study also found non-tumoral high uptake in pancreas caused by inflammation (mean SUV_max_ = 2.55) (**Figures 6A, B**). Seven surgical scars in our study showed high uptake of ^68^Ga-FAPI-04 (mean SUV_max_ = 3.34) (**Figures 6C, D**). Keloid is a fibroproliferative reticular dermal disorder characterized by inflammation, increased deposition of ECM protein, and invasion of the surrounding healthy skin (37). FAP expression is observed in keloid (37) and in the physiological process of wound healing (38). Consistent with its role in fibrosis, FAP has been found to be expressed in fibroblasts and hepatic stellate cells (HSCs) activated in cirrhosis but not in normal human livers (39, 40). Crohn’s disease is a chronic inflammatory bowel disease in which myofibroblasts play a key role in the process of fibrosis. It is worth mentioning that the myofibroblasts isolated from a colon specimen of a patient with stenosis were FAP positive. Tumor necrosis factor (TNF) and transforming growth factor (TGF) can further induce the expression of FAP (41). The systemic non-tuberculous mycobacterium granuloma case suggests that ^68^Ga-FAPI-04 PET can be used as an effective imaging tool to detect the degree of infection and evaluate the therapeutic effect.

Similar to findings of FDG, our study showed that regions with high ^68^Ga-FAPI-04 uptake did not necessarily represent malignancy. A previous FAPI study analyzed SUV_max_ of 28 different types of tumors (31) and reported that although ^68^Ga-FAPI-04 uptake was higher in malignant lesions than in benign lesions and physiological uptake regions, there was still some overlap. There was no statistically significant difference in SUV_max_ between the benign uptake regions and the physiological regions. This suggests that SUV_max_ cannot be used as a differential diagnostic index of physiological and benign uptake regions.

There were some limitations in our study. Since this study was retrospective, pathological verification of the lesions was challenging. Most of the diagnosis were based on the clinical history and the experience of the reviewers and with reference to other imaging modalities (CT, MRI, ultrasound, etc.), similar to previous studies. Although our sample size was relatively large, it was not possible to cover all non-tumor uptake regions. There is still a need to accumulate more cases in order to summarize the features of non-malignant lesion uptake in ^68^Ga-FAPI-04 as a way to improve the accuracy of diagnosis.

## Conclusions

This study evaluated the SUV_max_ of ^68^Ga-FAPI-04 in non-tumoral uptake regions with a relatively large sample population and elaborated the possible pathophysiological mechanisms of these non-tumoral uptake regions. The results indicated that quite a few tissues exhibit physiological uptake of ^68^Ga-FAPI-04. Gender has a more significant effect on physiological expression of ^68^Ga-FAPI-04 than age. No statistical differences in ^68^Ga-FAPI-04 uptake were found between benign and physiological high uptake regions. Our study showed that regions with high ^68^Ga-FAPI-04 uptake did not necessarily represent malignancy, and therefore, being familiar with physiological ^68^Ga-FAPI-04 uptake and the uptake of typical benign lesions can be helpful for physicians to interpret images and diagnose disease.

## Data availability statement

The original contributions presented in the study are included in the article/Supplementary Material. Further inquiries can be directed to the corresponding author.

## Ethics statement

Written informed consent was obtained from the individual(s) for the publication of any potentially identifiable images or data included in this article.

## Author contributions

NQ participated in its design and coordination and drafted the manuscript. HW conducted statistical processing on the data. HYW and SR reviewed the images. ZY and XC contributed to data collection. YG, FX, FH, and JZ provided critical review and substantially revised the manuscript. All authors read and approved the final manuscript.

## Funding

The study was partially supported by the National Natural Science Foundation of China (81871388), Project of Science and Technology Commission of Shanghai Municipality (19DZ1930703).

## Acknowledgments

We would like to thank Qiaoyi Xue (Central Research Institute, UIH Group, Shanghai, China) for her linguistic assistance and her substantial revision during the preparation of this manuscript.

## Conflict of interest

The authors declare that the research was conducted in the absence of any commercial or financial relationships that could be construed as a potential conflict of interest.

## Publisher’s note

All claims expressed in this article are solely those of the authors and do not necessarily represent those of their affiliated organizations, or those of the publisher, the editors and the reviewers. Any product that may be evaluated in this article, or claim that may be made by its manufacturer, is not guaranteed or endorsed by the publisher.
